# Clozapine-induced pyoderma gangrenosum. A case report

**DOI:** 10.1192/j.eurpsy.2024.328

**Published:** 2024-08-27

**Authors:** M. Peyioti, P. Argitis, O. Pikou

**Affiliations:** ^1^Psychiatric; ^2^Dermatology, General Hospital of Corfu, Corfu, Greece

## Abstract

**Introduction:**

Clozapine is an atypical antipsychotic medication which is mainly used in cases of treatment-resistant schizophrenia. Although it has several advantages over other typical and atypical antipsychotic medication, such as fewer relapses and lowering the risk of tardive dyskinesia and suicide it also has a range of adverse effects which makes compliance an issue for many patients.

Pyoderma gangrenosum (PG) is a rare neutrophilic dermatosis clinically characterized by painful pustules or nodules that rapidly evolve in ulcers with violaceous, undermined borders and raised periphery. The etiopathogenesis of the disease remains unclear, however PG is usually manifested in the setting of an underlying immune-mediated disease, more commonly inflammatory bowel disease, rheumatoid arthritis and haematological malignancies.

**Objectives:**

Nevertheless, in the literature there are scarce reports of drug-induced PG.

**Methods:**

We present the case of a 56-year-old woman with a diagnosis of refractory schizophrenia on clozapine treatment for 4 months, who was admitted to the emergency department for a skin eruption localized on the trunk, genital area and extremities. The clinical examination revealed numerous, discrete erythematous macules, papules and plaques with central necrosis, and multiple, sharply marginated ulcers with undermined, red to purple border. Further physical examination disclosed no systemic findings and laboratory analyses and skin biopsy were performed.

**Results:**

On work-up, white blood cell count and C-reactive protein (CRP) were elevated, while blood and pustule cultures were negative. Histologic examination revealed dermal necrosis and inflammation, features consistent with the diagnosis of PG. The pathergy test was positive.

**Image:**

**Figure:**
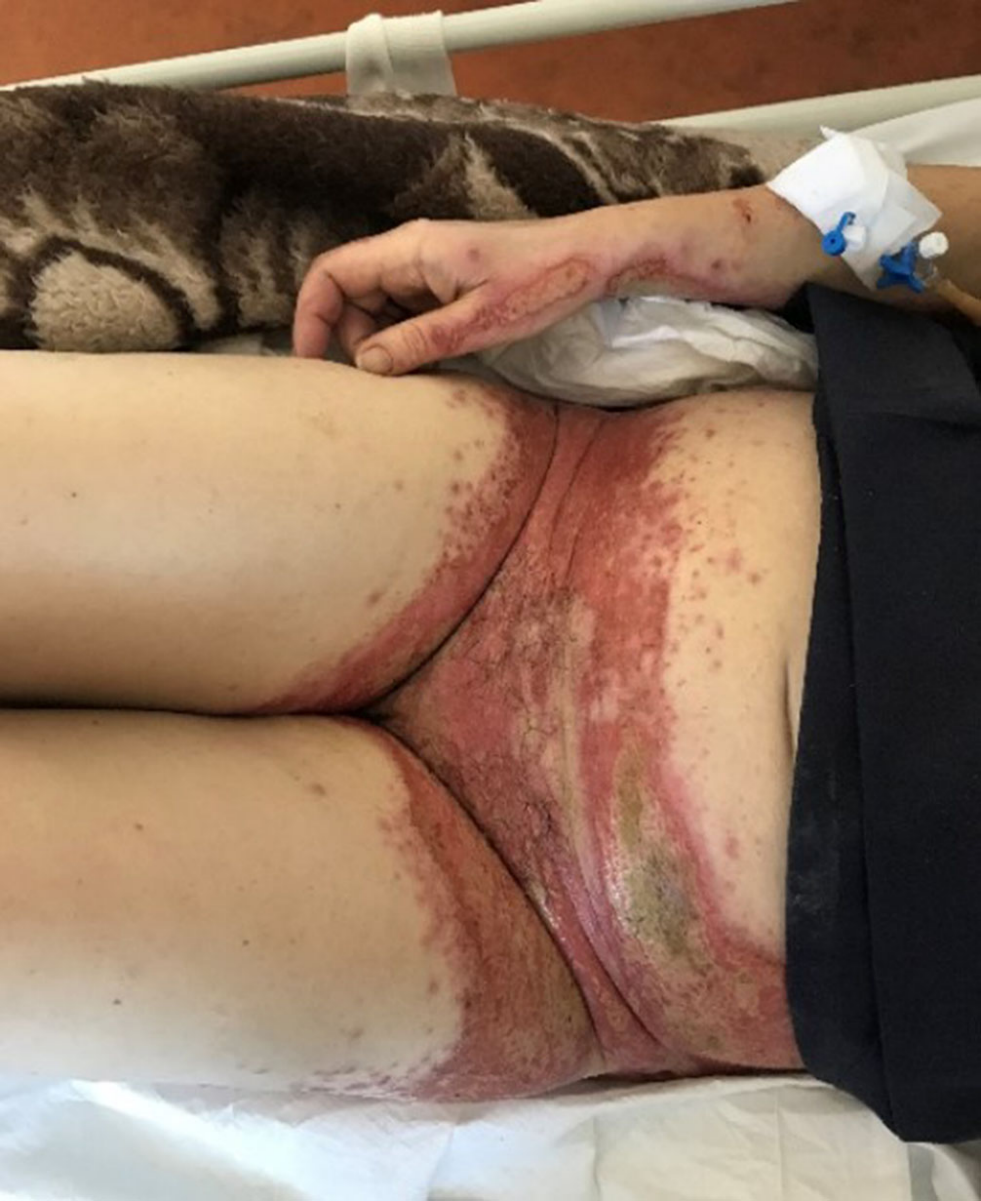

**Image 2:**

**Figure:**
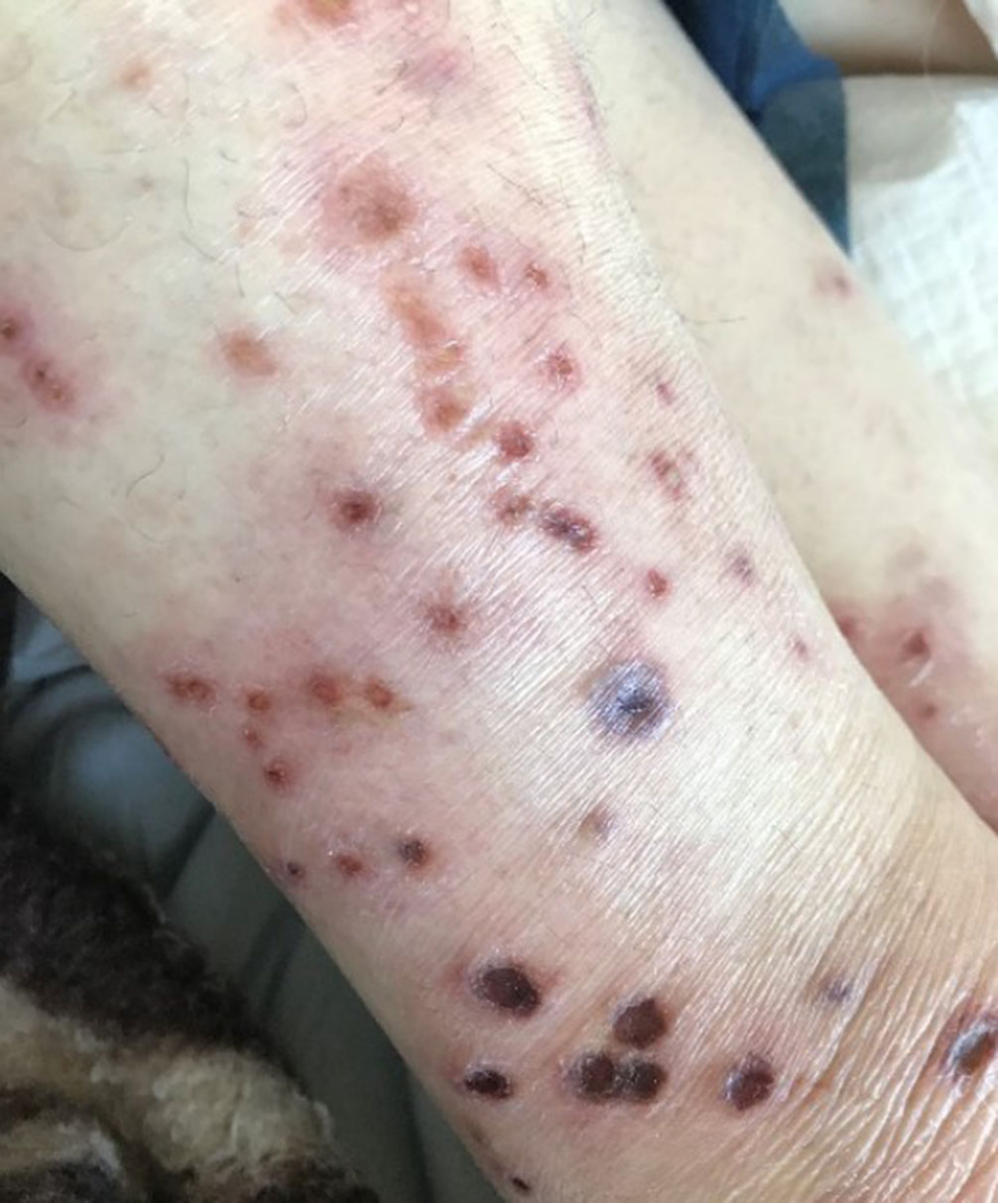

**Image 3:**

**Figure:**
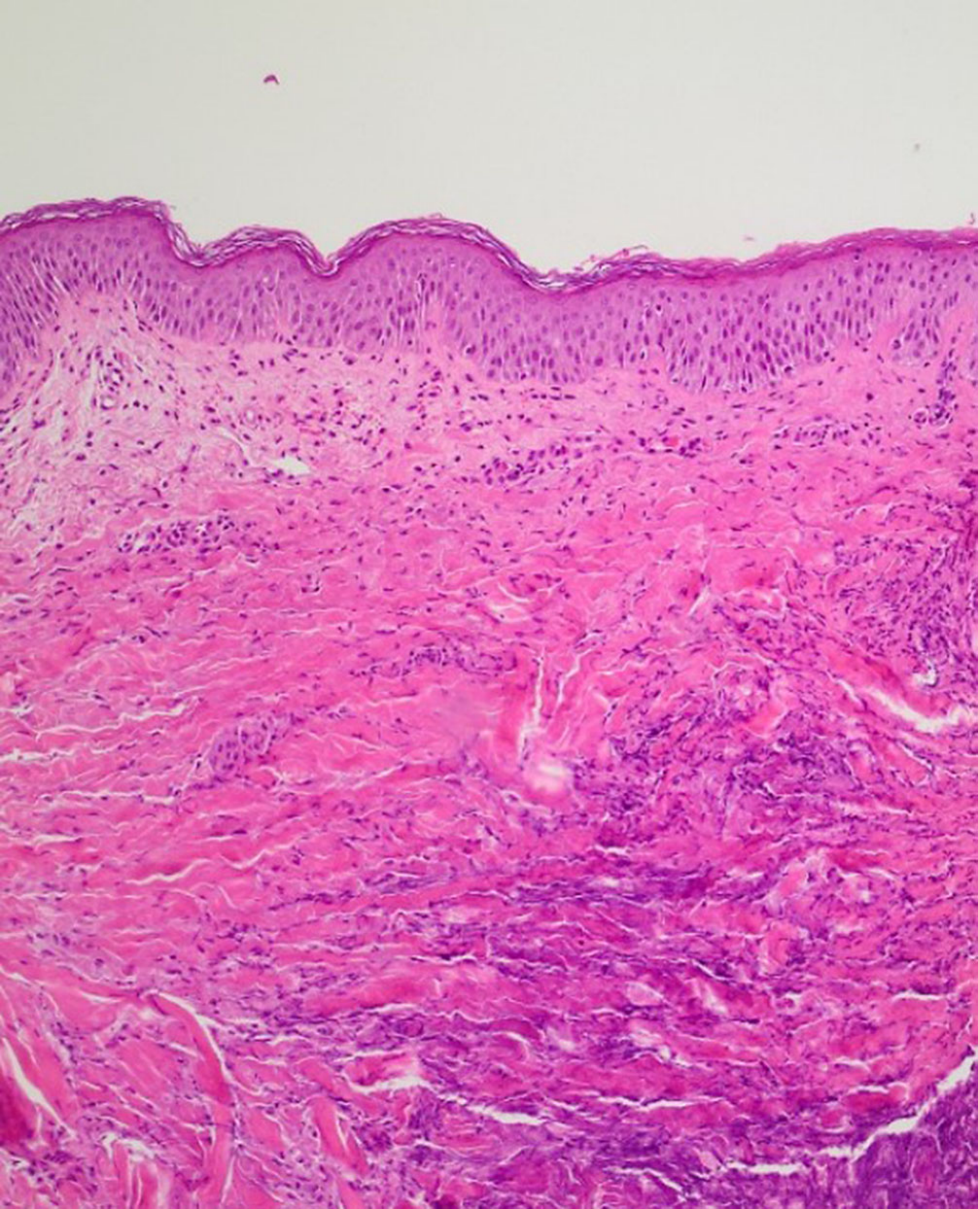

**Conclusions:**

Pyoderma gangrenosum is a rare autoinflammatory skin disorder of unknown etiology. The pathogenesis of the disease is not well understood, but drug-induced PG is considered to result from neutrophil dysfunction and dysregulation of the inflammatory response. Cases of drug-induced PG are rare and attributed to certain drugs. Clozapine is an antipsychotic medication for the treatment of refractory schizophrenia. It is suggested that clozapine alters plasma TNF-α levels and thus can modulate the inflammatory response. To date, a variety of adverse skin reactions (Stevens- Johnson syndrome, DRESS syndrome etc) have been previously described in the literature. However, to the best of our knowledge, this is the first case which strongly indicates the likely association between clozapine and drug-induced PG.

**Disclosure of Interest:**

None Declared

